# The landscape of the long non-coding RNAs and circular RNAs of the abdominal fat tissues in the chicken lines divergently selected for fatness

**DOI:** 10.1186/s12864-022-09045-y

**Published:** 2022-12-01

**Authors:** Yang Jing, Bohan Cheng, Haoyu Wang, Xue Bai, Qi Zhang, Ning Wang, Hui Li, Shouzhi Wang

**Affiliations:** 1 Key Laboratory of Chicken Genetics and Breeding, Ministry of Agriculture and Rural Affairs, Harbin, 150030 People’s Republic of China; 2 Key Laboratory of Animal Genetics, Breeding and Reproduction, Education Department of Heilongjiang Province, Harbin, 150030 People’s Republic of China; 3 College of Animal Science and Technology, Northeast Agricultural University, Harbin, 150030 People’s Republic of China

**Keywords:** Long non-coding RNA, Circular RNA, Competing endogenous RNA network, Fat deposition, Chicken

## Abstract

**Background:**

Excessive deposition of abdominal fat poses serious problems in broilers owing to rapid growth. Recently, the evolution of the existing knowledge on long non-coding RNAs (lncRNAs) and circular RNAs (circRNAs) have established their indispensable roles in multiple physiological metabolic processes, including adipogenesis and fat deposition. However, not much has been explored on their profiles in the abdominal fat tissues of broilers to date. In the study, we aimed to characterize the vital candidates of lncRNAs and circRNAs and their underlying regulations for abdominal fat deposition in broilers.

**Results:**

The present study sequenced the lncRNAs and circRNAs expression profiles in the abdominal fat tissues isolated from 7-week-old broilers, who were divergently selected for their fatness. It identified a total of 3359 lncRNAs and 176 circRNAs, demonstrating differential expressed (DE) 30 lncRNAs and 17 circRNAs between the fat- and lean-line broilers (|log2FC| ≥ 1, *P* < 0.05). Subsequently, the 20 cis-targets and 48 trans-targets of the candidate DE lncRNAs were identified for depositing abdominal fat by adjacent gene analysis and co-expression analysis, respectively. In addition, the functional enrichment analysis showed the DE lncRNAs targets and DE circRNAs host genes to be mainly involved in the cellular processes, amino/fatty acid metabolism, and immune inflammation-related pathways and GO terms. Finally, the vital 16 DE lncRNAs located in cytoplasm and specifically expressed in fat/lean line and their targets were used to construct the lncRNA-miRNA-mRNA competing endogenous RNA (ceRNA) regulatory network, comprising 7 DE lncRNAs, 28 miRNAs, 11 DE mRNAs. Notably, three lncRNAs including XR_001468036.2, XR_003077610.1 and XR_001466431.2 with the most connected degrees might play hub regulatory roles in abdominal fat deposition of broilers.

**Conclusions:**

This study characterized the whole expression difference of lncRNAs and circRNAs between the two lines broilers with divergently ability of abdominal fat. The vital candidate DE lncRNAs/circRNAs and ceRNA regulations were identified related to the deposition of abdominal fat in chicken. These results might further improve our understanding of regulating the non-coding RNAs in obesity.

**Supplementary Information:**

The online version contains supplementary material available at 10.1186/s12864-022-09045-y.

## Background

Obesity is a multifactorial metabolic disorder characterized by the deposition of abnormal or excessive fat. Obesity poses a serious threat to public health by triggering multiple chronic diseases, like the fatty liver, type 2 diabetes, cardiovascular disease, and several cancers [1]. Considering the unprecedented increase and prevalence of obesity worldwide both in humans as well as livestock, there is a pressing need for investigations to reveal the underlying factors of pathogenesis for mitigating and preventing obesity [2, 3]. Although the extant studies have attempted in identifying the causative factors of obesity like diet and genetics [4], they have not been able to explain the effects sufficiently. Recent studies have indicated epigenetic factors like the non-coding RNAs (ncRNAs) to be associated with obesity [5]. Hence, revealing the landscape of the ncRNAs in the adipose tissues can further promote the identification of causative factors of obesity.

Growing evidence indicates that ncRNAs have important functions in regulating certain phenotypes in animals like the traits associated with fat deposition. Long non-coding RNAs (lncRNAs) are ncRNAs comprising transcripts having a nucleotide length beyond 200 that are unable to be translated into proteins. LncRNAs show ubiquitous expression in various tissues with reported roles in multiple biological processes, like transcript stability, promoter-specific gene regulation, epigenetic modification, and imprinting mark [6]. Numerous studies in mice and humans have identified the lncRNAs to be closely associated with obesity. Prarl1, a lncRNA detected in human white adipose tissues, is reportedly involved in differentiating the adipocytes by their interaction with RBM12/NCoAA [7]. Similarly, the lncRNAs, like lnc-U90926 [8], AdipoQ AS lncRNA [9], and GAS5 [10], were down-regulated in the obese mice, inhibiting adipogenesis. Besides, the lncRNAs Meg3 [11], Plnc1 [12], and PVT1 [13] having vital roles in promoting adipogenesis through the Wnt/β-catenin and PPAR signaling pathways were up-regulated in the obese individuals. Circular RNAs (circRNAs) formed by back splicing from the 3′ and 5′ ends of the linear RNAs possess a covalently closed continuous loop [14]. CircRNAs were previously considered as a class of non-coding RNAs. Until 2017, Pamudurti et al. identified a group of circRNA that could translate by using the start codon of host mRNA in the fly head, which proved that some of the circRNAs could encode proteins [15]. Owing to the few studies on circRNAs, numerous functions of the circRNAs functions are elusive. The circRNAs identified so far have been reported to usually function as the microRNA sponges and transcription regulators [16–18]. Several critical candidate circRNAs have been identified in pig [19], mouse [20] and human [21] to be associated with adipogenesis. They have demonstrated differential expressions in the fat tissues in the obese vs control individuals. The underlying mechanism might be associated with their critical role in regulating preadipocyte differentiation [22]. However, the expression profiles of the lncRNAs and circRNAs in the abdominal fat tissues from obese individuals have been rarely studied.

Chicken is an interesting model organism for studying obesity, owing to its unique metabolic features, like hyperglycemia, insulin resistance [23]. Our group has successfully constructed broiler lines that were divergently selected for their abdominal fat traits [24], serving as an ideal model for investigating the pathogenesis of obesity. To reveal the roles of lncRNAs and circRNAs in the abdominal fat deposition (AFD) of the broilers, the expression landscape of these lncRNAs and circRNAs were profiled and the candidate regulators were identified based on the fat-and lean-line broilers. The results of the present study might therefore, offer comprehensive insights and reference into the roles of the lncRNAs and circRNAs regulating the deposition of abdominal fat in the broilers as well as other animals.

## Results

### The lncRNA and circRNA profiling in the abdominal fat tissues from the broiler

**Fig. 1:**
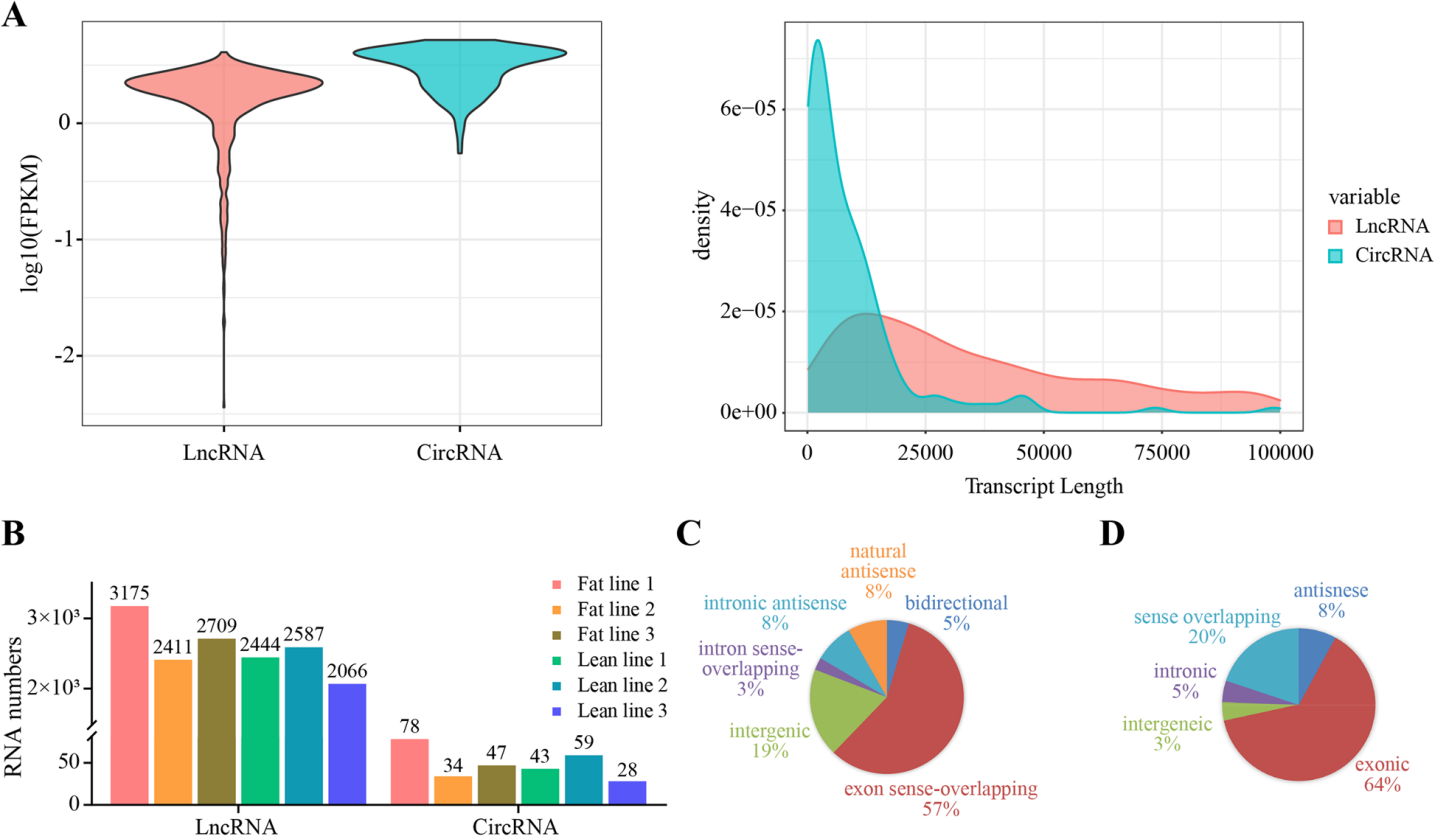
Characterization of the lncRNAs and circRNAs in the broiler abdominal fat tissues. **A** The whole expression levels and length distribution of lncRNAs and circRNAs. **B** The numbers of lncRNAs and circRNAs were identified in all the samples. **C** The classification and proportion of the identified lncRNAs. Exon sense-overlapping: the exon of the lncRNAs overlapping a coding transcript exon on the same genomic strand; intron sense-overlapping: the lncRNA overlapping the intron of a coding transcript on the same genomic strand; intronic antisense: the lncRNA overlapping the intron of a coding transcript on the antisense strand; natural antisense: the lncRNA transcribed from the antisense strand and overlapping with the exon of a coding transcript; bidirectional: the lncRNA oriented in a head to head fashion to a coding transcript within 1000 bp; intergenic: there are no overlapping or bidirectional coding transcripts near the lncRNA. **D** The classification and proportion of the identified circRNAs. Exonic: the circ-RNAs sourced from exons; intronic: the circ-RNAs sourced from introns; intergenic: the circRNAs sourced from intergenic regions; sense overlapping: the circRNAs sourced from the exons and intergenic regions; antisense: the circRNAs sourced from the exons and introns

In the present study, the Ribo-zero RNA-Seq was performed using the abdominal fat tissues from six broilers (3 of fat line and 3 of lean line). After adaptor-trimming and removing the low-quality read, a total of 514 million high-quality clean reads were obtained having a Q30 rate of over 90% (Table 1). Subsequently, the clean reads were mapped to the chicken reference genome (galGal6) and the lncRNAs and circRNAs were then identified and analyzed. The genome mapping rate were summarized in Table 1. The mapping rate of circRNAs ranged between 63.99–64.96%, whereas the mapping rate of lncRNAs ranged between 87.58–89.95%. Consistently, the circRNAs have less occupancy in the transcriptome than the lncRNA and a total of 3359 lncRNAs and 176 circRNAs were identified in the six samples. The violin plot showed that the expression level of circRNAs was slightly higher than that of lncRNAs, and most lncRNAs lengths were close to 12,500 bp and most circRNAs lengths were about 4000 bp (Fig. 1A). The detailed numbers of lncRNAs and circRNAs in each sample were shown in Fig. 1B. Amongst the identified lncRNAs, the majority were the exon sense-overlapping (57%), followed by the intergenic ones (19%) (Fig. 1C; Additional file 2: Fig. S1). For the circRNAs, most of them were generated from the exonic regions (64%), followed by the sense overlapping regions (20%) and antisense regions (8%) (Fig. 1D; Additional file 2: Fig. S1). The expression of lncRNAs and circRNAs were subsequently calculated. The Principal Component Analysis indicated a difference in the expression profiles of the lncRNAs and circRNAs between the fat- and lean-line broilers, though the variation was lesser in the circRNAs than the lncRNAs (Additional file 2: Fig. S2).

**Table 1:** Statistics of sequence reads and mapping to reference genome

Sample Name	Raw reads	Clean reads	Q30	CircRNA Mapping rate	LncRNA Mapping rate
F-1.Input	89,018,462	88,610,072	91.16%	64.23%	89.95%
F-2.Input	70,672,538	70,347,050	90.55%	63.99%	87.58%
F-3.Input	83,208,062	82,791,982	91.09%	64.10%	88.91%
L-1.Input	95,689,358	95,286,156	91.18%	64.96%	88.23%
L-2.Input	92,148,032	91,859,392	92.10%	64.69%	89.45%
L-3.Input	85,861,286	85,559,974	92.26%	64.06%	88.46%

### Identification of the differentially expressed lncRNAs and circRNAs between the fat- and lean-line broilers

**Fig. 2:**
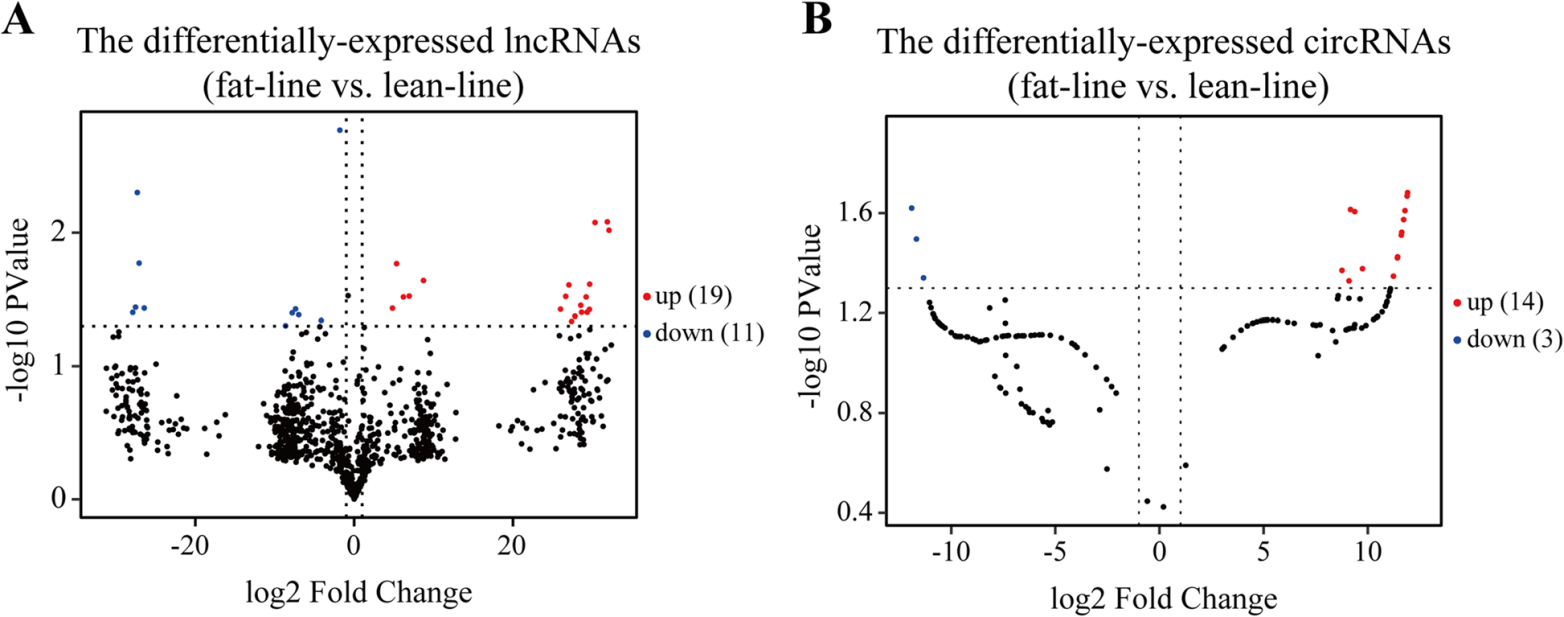
The identification of the differentially expressed lncRNAs and circRNAs between the fat- and lean-line broilers. **A** The volcano plot of the differentially-expressed lncRNAs (fat-line vs. lean-line). **B** The volcano plot of the differentially-expressed circRNAs (fat-line vs. lean-line)

The expression profiles of the lncRNAs and circRNAs in the abdominal fat were further compared between the fat- and lean-line broilers. For this, the differentially expressed (DE) lncRNAs and circRNAs were screened using the cuffdiff (v2.1.1) and edgeR software (v3.16.5), respectively. The lncRNAs with FPKM value of > 0 in more than 2 samples in either group were screened for the DE analysis. Due to the fewer number of identified circRNAs in our samples, all the circRNAs with a CPM value of > 0 were used for analyzing the DE RNAs. Finally, 30 lncRNAs (19 up-regulated and 11 down-regulated, fat-line vs. lean-line) and 17 circRNAs (14 up-regulated and 3 down-regulated, fat-line vs. lean-line) were found to differentially express in the abdominal fat between the two lines of broilers (|log2FC| ≥ 1, *P*-value < 0.05) (Fig. 2; Additional file 1: Tables S1 and S2; Additional file 2: Fig. S1). Among these DE lncRNAs, 14 lncRNAs were only expressed in the fat line, while 5 lncRNAs were merely expressed in the lean line. In the case of the DE circRNAs, 9 circRNAs were only expressed in the fat line, while 3 circRNAs were merely expressed in the lean line. Three each of the DE lncRNAs and circRNAs were randomly selected to validate the expression difference between the two lines. The results showed a high consistency between qRT-PCR and RNA-seq results (Additional file 2: Fig. S3), which demonstrated the reliability of DE lncRNAs and circRNAs identification in the present study. The distinguishable expression patterns of the DE lncRNAs and circRNAs in the abdominal fat between the two lines broilers suggested that the differential deposition of abdominal fat in the two lines broilers might be caused by the regulation of the DE lncRNAs and circRNAs.

### Construction of the co-expression network and target prediction

**Fig. 3:**
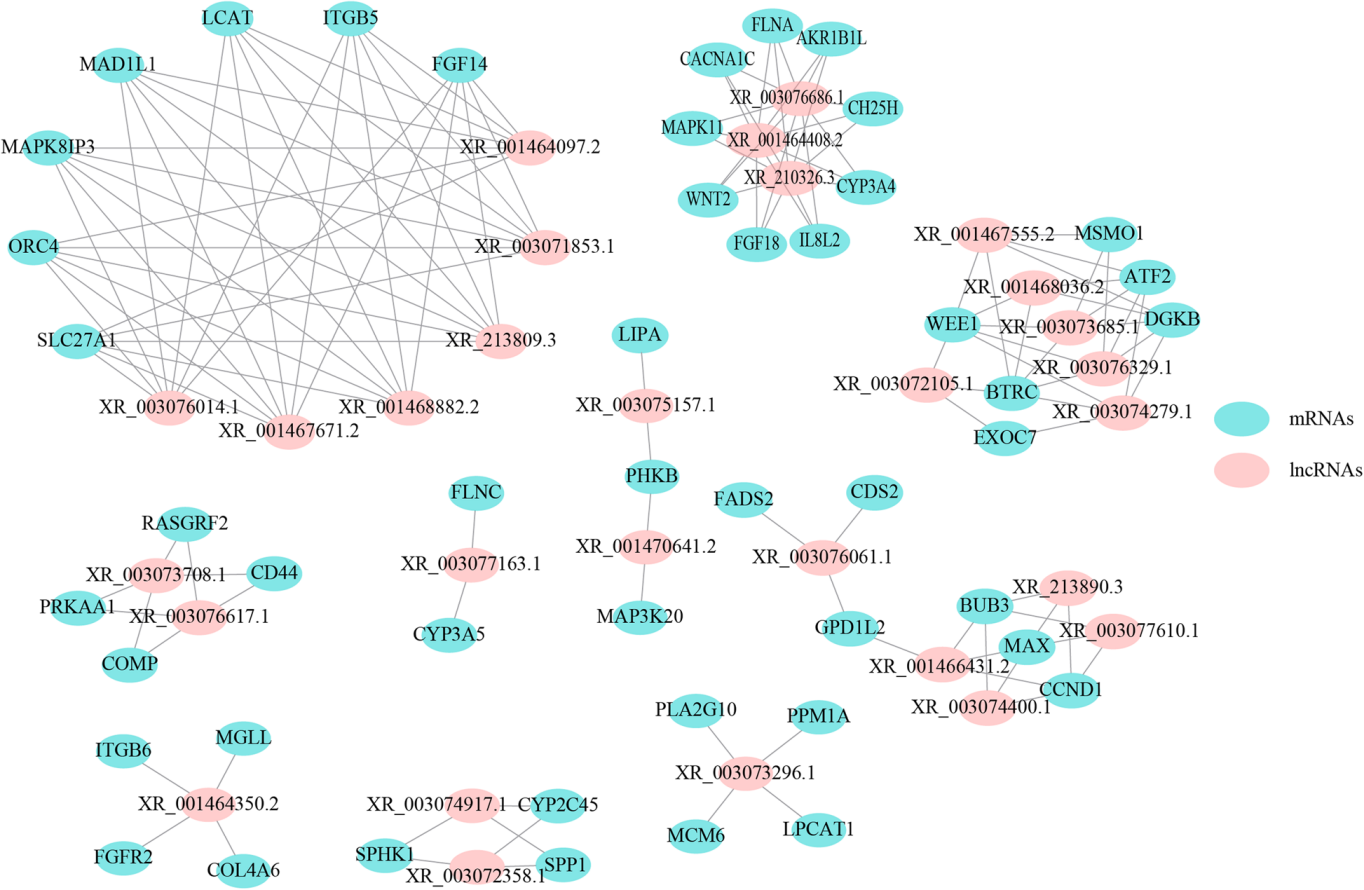
The co-expression network for the differentially expressed lncRNAs and their potential target mRNAs. The Pearson correlations (absolute value > 0.95 and FDR < 0.05) between the expression of the lncRNAs and mRNAs were performed to construct the relations network using the Cytoscape software (version 3.6.1). Ellipses with pink color are lncRNAs, while light blue represents the mRNAs

To further explore the regulation of lncRNAs in the deposition of abdominal fat, the DE mRNAs (previously identified) annotated in the lipid metabolism and cellular process-related pathways (Additional file 1: Table S3) and the DE lncRNAs identified in the present study were selected for constructing the mRNA-lncRNA co-expression network. A total of 48 mRNAs and 29 lncRNAs comprising 140 connections were found in the network (Fig. 3; Additional file 1: Table S4; |cor| > 0.95, FDR < 0.05). Each mRNA was found to be regulated by multiple lncRNAs, and each lncRNA was also found to be correlated with more than one mRNAs. Interestingly, six lncRNAs were all found to co-express with the genes *ORC4*, *MAPK8IP3,* and *LCAT* involved in the “cell cycle”, “MAPK signaling pathway” and “Glycerophospholipid metabolism”, respectively. Additionally, three lncRNAs including XR_001467555.2, XR_003073685.1, and XR_003076329.1 identified to co-express with the steroid biosynthesis-related gene (*MSMO1*) and cell cycle-related gene (*WEE1*) (Fig. 3; Additional file 1: Table S4). These results demonstrated that the DE lncRNAs could affect the deposition of abdominal fat by regulating the genes related to lipid metabolism and cellular processes. CircRNAs are conventionally known to function as miRNA sponges. As a difference in the construction of the library, the miRNA expression was not profiled in the present study. Alternatively, the potential miRNA targets and involved interactions were predicted for the circRNAs using the online tools of miRDB (http://www.mirdb.org/), yielding 364 potential binding relations between 17 circRNAs and 314 miRNAs (Additional file 1: Table S5).

### Functional enrichments of the DE lncRNAs targets and circRNAs host genes between the fat-and lean-line broilers

**Fig. 4:**
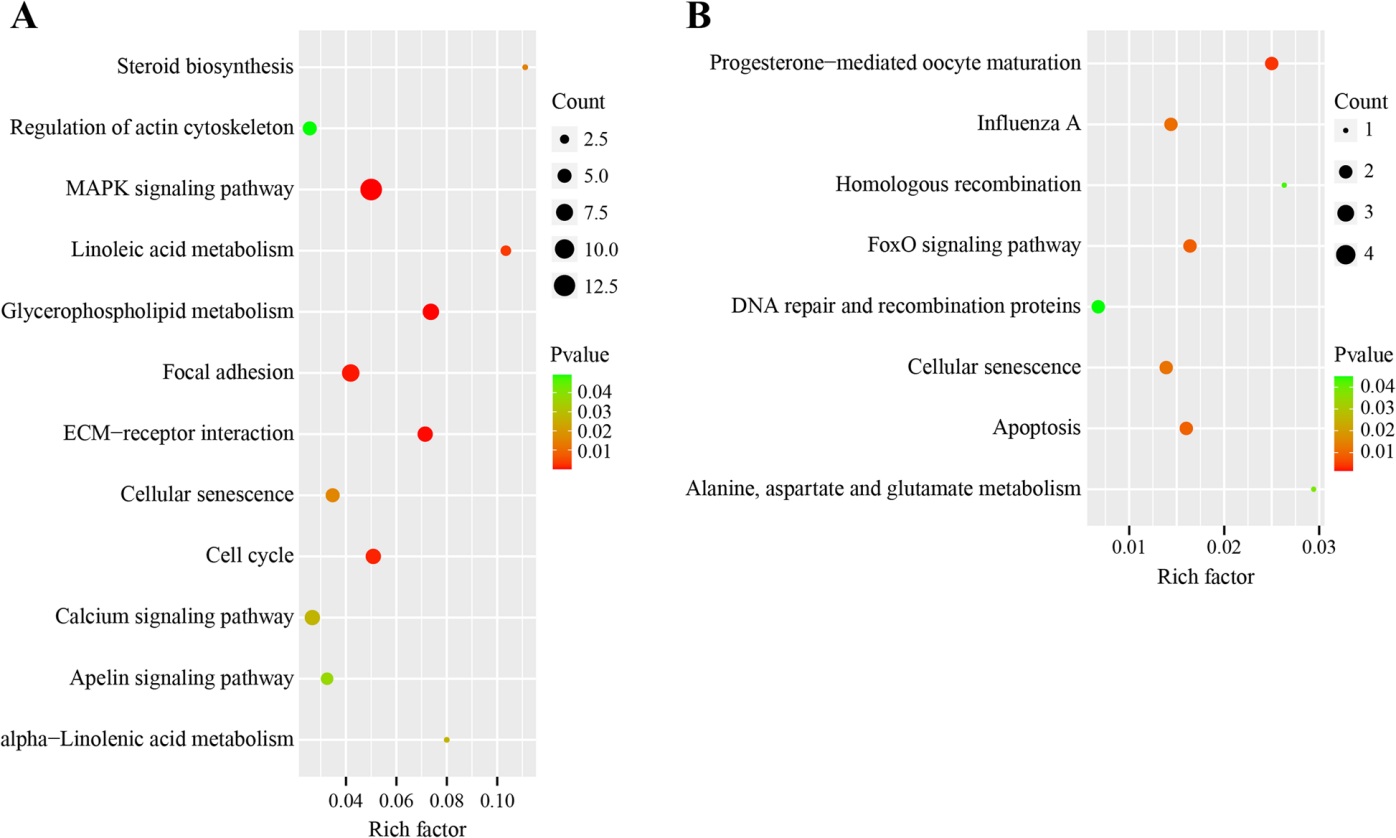
The KEGG pathways enrichment for differentially expressed lncRNAs targets (**A**) and circRNAs host genes (**B**). Y-axis stands for pathways, and X-axis is a rich factor (differentially expressed genes numbers enriched in the pathway/ all background genes numbers in this pathway). The bubble size and color represent the enriched genes numbers and enriched significance, respectively. The enrichment analysis was performed by R using the latest KEGG pathways files of chicken

To explore the potential functional variations, the DE lncRNAs targets and circRNAs host genes were subsequently annotated by the KEGG and GO database. The KEGG pathway enrichment revealed the DE lncRNAs-associated genes to be enriched in 54 signaling pathways, among which 12 pathways were significantly enriched (Additional file 1: Table S6). The significantly enriched pathways mainly included the lipid metabolism-related pathways, like those of the “Steroid biosynthesis”, “Glycerophospholipid metabolism”, “Linoleic acid metabolism” and “MAPK signaling pathway”, and cell process-related pathways, like those of the “Cellular senescence” and “Cell cycle” (Fig. 4A). These enrichments pathways demonstrated that the DE lncRNAs had a significant influence on the abdominal adipogenesis and lipid metabolism in the fat- and lean-line broilers. For the KEGG enrichment of the DE circRNAs-associated genes, a total of 36 signaling pathways were found to be enriched, among which 8 pathways reached significant levels (Additional file 1: Table S7). The significantly enriched pathways were mainly found to be associated with the cellular processes and metabolism, including the “FoxO signaling pathway”, “Apoptosis” and “Cellular senescence” (Fig. 4B). Moreover, the DE circRNAs-associated genes annotated the other pathways involved in the cellular processes and lipid metabolism, such as the “VEGF signaling pathway”, “p53 signaling pathway”, “TGF-beta signaling pathway”, “Adipocytokine signaling pathway”, “Insulin signaling pathway” and “Wnt signaling pathway” (Additional file 1: Table S7). These enriched pathways reflected the important roles of the circRNAs in regulating the adipocyte growth, differentiation, and apoptosis of the abdominal fat tissues in the fat- and lean-line broilers. The GO enrichments described the functions of the DE lncRNAs and circRNAs-associated genes. Consequently, the main significant enriched GO terms of the biological process were found to be related to the cellular process and metabolism for both the DE lncRNAs and circRNAs-associated genes (Additional file 2: Fig. S4; Additional file 1: Table S8 and Table S9).

### Analysis of the lncRNA-miRNA-mRNA ceRNA regulatory network

**Fig. 5:**
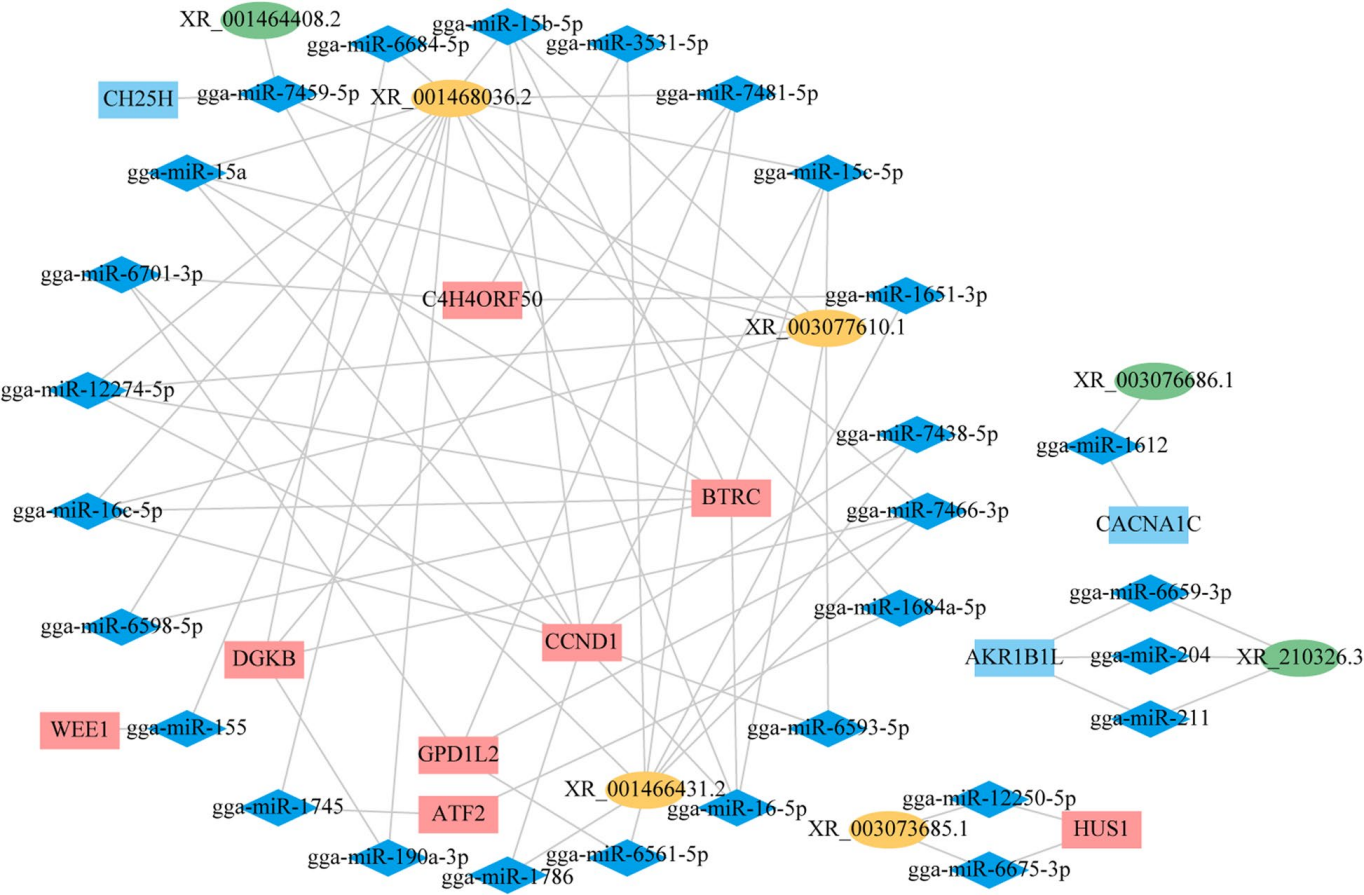
Construction of the lncRNA-miRNA-mRNA ceRNA regulatory network. The differentially expressed (DE) lncRNAs that were specifically expressed in either of one group were chosen for constructing the global ceRNA network using the Cytoscape software (version 3.6.1). The ceRNA network contained 7 DE lncRNAs, 28 miRNAs, and 11 DE mRNAs. Ellipse, up-regulated lncRNAs (orange), down-regulated lncRNAs (green); blue rhombus, miRNAs; rectangle, up-regulated mRNAs (pink), down-regulated mRNAs (blue); Fat line vs. lean line

To explore the underlying regulated associations between the DE lncRNAs and their target mRNAs, the lncRNA-miRNA and miRNA-mRNA pairs were predicted using online tools of miRDB. Considering important roles of specifically expressed DE lncRNAs, the specifically expressed DE lncRNAs of any one group that located in cytoplasm were screened for building the ceRNA network. A total of 7 DE lncRNAs, 28 miRNAs, 11 DE mRNAs, and 75 edges were identified in the ceRNA network (Fig. 5; Additional file 1: Table S10). In the ceRNA network, three lncRNAs including XR_001468036.2, XR_003077610.1 and XR_001466431.2 with the most connected degrees might play hub regulatory roles. For lncRNA XR_001468036.2 sub-network, it contained 14 miRNAs, 6 mRNAs and 36 edges; for lncRNA XR_003077610.1 sub-network, it included 8 miRNAs, 3 mRNAs and 23 edges; for lncRNA XR_001466431.2 sub-network, it was comprised of XR_001466431.2, 8 miRNAs, 4 mRNAs and 19 edges.

## Discussion

Obesity characterized by the excessive deposition of body fat has gained increasing prevalence worldwide. It is commonly related to multiple chronic diseases, like hypertension, steatohepatitis, cardiac diseases, and even cancer [25, 26]. In-depth research is attempting to reveal the underlying mechanism responsible for the occurrence and development of obesity. Recently, the non-coding RNAs like the lncRNAs and circRNAs have gained attention for studying obesity, since they play vital roles in adipogenesis and lipid metabolism both in mammals and poultry [19, 27, 28]. However, the whole landscape of lncRNAs and circRNAs in the fat tissues of obese and lean individuals has not yet been characterized. The Northeast Agricultural University broiler lines, divergently selected for the abdominal fat content (NEAUHLF) serve as an ideal model for genetic obesity research since it has over 10-fold differences in the AFP between the fat- and lean-line broilers in the 23rd generations [29]. In the present study, the RNA sequencing results of the lncRNAs and circRNAs were extracted from our previous study [30], and their expression profiles in the abdominal fat tissues of the fat- and lean-line broilers were investigated. To our knowledge, the study described the whole landscape of lncRNAs and circRNAs in abdominal fat tissues from broilers with divergently ability of AFD for the first time, and offered underlying candidates regulatory RNAs for AFD in chicken.

This study identified a total of 3359 lncRNAs and 176 circRNAs in the abdominal fat tissues from the fat- and lean-line broilers (detailed numbers of RNAs see in Fig. 1B). As evident, the lncRNA and circRNA expression were tissue- and period-specific [31, 32]. Even in the fat tissues, the numbers of lncRNAs and circRNAs identified varied among the different animals, such as 3716 lncRNAs [33] and 5141 circRNAs [34] identified in the subcutaneous fat of cattle, 9082 identified lncRNAs identified in the tail fat of sheep [35], and 1637 circRNAs identified in the chicken abdominal fat [36]. Coincidently, similar numbers of lncRNAs were identified in the broiler abdominal fat, but our study identified fewer numbers of circRNAs in the abdominal fat of 7 wks broilers than that in the other animals, possibly related to the various fat samples, species, and development stage. Interestingly, the majority of the lncRNAs and circRNAs in our study were the exon sense-overlapping and exonic, respectively (Fig. 1C and Fig. 1D; Additional file 2: Fig. S1). The exon sense-overlapping lncRNAs are viewed as mRNA transcript variants, with regulatory roles in the binary expression of the genes [37]. The exonic circRNAs on the other hand, function as miRNA sponges, or regulate the parental gene transcription, and interactions with the RNA-binding proteins [38]. Thus, the differential expressed pattern of the exon sense-overlapping lncRNAs and exonic circRNAs can be speculated to be associated with the various AFD between the broilers of the two lines.

The expression profiles of the lncRNAs and circRNAs were further compared in the abdominal fat tissues between the two lines, identifying 30 DE lncRNAs and 17 DE circRNAs (Fig. 2). The obesity-associated lncRNAs were found to have increased expression in the obese mice and were found to regulate the marker genes involved in the cell cycle pathways [39]. Sun et al. also identified more upregulated DE lncRNAs (213 of 249) in the blood of the obese participants, compared with those of the non-obese participants [40]. These enriched lncRNAs in obese individuals were proven to promote the entry of the cell into the mitotic clonal expansion phase, benefitting the early stages of adipogenesis [41]. Our results were consistent with these studies, where more upregulated DE lncRNAs were identified in the fat-line broilers. The circRNAs can regulate lipid metabolism and deposition by combining with the target miRNAs. In the two kinds of pigs with variable subcutaneous fat content, the DE circRNAs were identified and their targets were found to be enriched in the adipocyte differentiation, lipid metabolism, and disease-related pathways [42]. During mouse adipocyte differentiation, most candidate circRNAs were found to be significantly upregulated with their co-expressed liner transcripts, but the circRNAs were generally down-regulated in the high fat diet induced obesity, probably due to their association with the activation of the inflammatory pathways in the mature adipocytes [43]. Inconsistently, more up-regulated DE circRNAs were identified in the abdominal fat tissues of the fat-line broilers. Unlike high fat diet induced obesity, our genetic obese broilers demonstrated different regulated patterns of circRNAs in obesity and the fewer identified circRNAs than the other studies might be related to the persistent low inflammatory response caused by the deposition of the abdominal fat in our broilers. Based on the obese broiler model, our study identified the DE lncRNAs and circRNAs, which could be important for regulating fat deposition in the broilers.

To reveal the potential regulatory functions of the DE lncRNAs and circRNAs, their targets and host genes were subsequently utilized for performing the KEGG and GO enrichment analysis, respectively. The DE lncRNAs targets were found to be mainly involved in lipid metabolism and cell growth (Fig. 4A). Our results were similar to the studies in obese mice and pigs, demonstrating that the lncRNAs could regulate the genes involved in lipid metabolism and cellular cycles for facilitating adipogenesis [28, 39, 41]. Besides, the mRNAs profiles of the same samples were integrated with the DE lncRNAs profiles for constructing the co-expression networks. The results showed each mRNA to be regulated by multiple lncRNAs involved in lipid metabolism and cellular process, and each lncRNA was also found to be correlated with more than one mRNAs (Fig. 3). Importantly, the *LCAT* gene was trans-regulated by XR_001464097.2, XR_003071853.1, XR_213809.3, XR_001468882.2, XR_001467671.2 and XR_003076014.1 (Fig. 3; |cor| > 0.95, FDR < 0.05). The *LCAT* (*lecithin-cholesterol acyltransferase*) gene can catalyze the transacylation of the *sn*-2 fatty acid of lecithin to the free 3′-hydroxyl group of cholesterol in the high density lipoproteins (HDLs), promoting the reverse cholesterol transport [44]. The *LCAT* gene polymorphism has been proven to increase the risk of dyslipidemia and coronary atherosclerotic heart disease by decreasing the plasma high density lipoprotein cholesterol (HDL-C) levels in humans [45]. In mice, the transient overexpression of the *LCAT* gene can reduce macrophage accumulation, oxidation of LDL, and atherosclerosis [46]. The RNA-Seq analysis of the abdominal fat in the broiler revealed that the *LCAT* gene was significantly down-regulated in the low feed efficiency broiler group with more fat deposition, causing deficient HDL formation and interrupting the reverse cholesterol transport [47]. The *LCAT* gene was significantly up-regulated in the fat-line broilers (Additional file 1: Table S3) and was in line with the previous reports stating that the fat-line broilers possess higher plasma HDL-C levels than the lean-line broilers, resulting in a stronger ability to synthesize and transport triglycerides [48]. Thus, an increased expression of the *LCAT* gene can be speculated to be regulated by these six lncRNAs, partially contributing to an increase in the plasma HDL-C levels and leading to excessive deposition of abdominal fat in the fat line. Besides, the genes involved in the cellular cycle (*ORC4* and *WEE1* gene) were identified to co-express with the corresponding lncRNAs. OCR4 is one of the six subunits of the origin recognition complex (OCR), responsible for DNA replication by remodeling the structure of the origin of replication in humans [49]. The tumor suppressor, WEE1 regulates the replication forks and genome stability, inhibiting the replication of cells with altered DNA in humans [50]. In our study, *OCR4* and *WEE1* were found to be significantly up-regulated in the fat-line broilers. Although there are fewer reports of these two genes in obesity, it can be presumed that their differential expression regulated by the lncRNAs can affect the deposition of abdominal fat in the broilers by regulating the preadipocyte proliferation.

LncRNAs reportedly serve as the ceRNA for regulating the differentiation of adipocytes in mammals by sponging the miRNAs [51, 52]. Recently, the lncRNA-associated ceRNA network was reportedly involved in the differentiation of the chicken preadipocytes, identifying 251 ceRNAs relationships [53]. The present study selected 16 DE lncRNAs that were specifically expressed in either of the group and located in the cytoplasm and their target mRNAs for building the lncRNA-miRNA-mRNA ceRNA regulatory network in the AFD of broiler. The three hub lncRNAs with connected degrees of more than 7 were identified in ceRNA network (Fig. 5). XR_001468036.2 is an intergenic lncRNA (lincRNA), which only expressed in abdominal fat tissues of fat line broilers. As a kind of common lncRNAs, lincRNAs are characterized as independent transcribed lcnRNAs without any overlaps with coding genes. In humans, the lincRNAs occupied over half of lncRNAs transcripts, which suggested indispensable roles in various physiological processes [54]. Studies have identified important lincRNAs involved in regulating cell differentiation, migration and pluripotency [55–57]. Zou et al. have revealed the important lincRNAs in regulating intramuscular fat of pig [58]. In the present study, the lincRNA XR_001468036.2 might regulate lipid metabolism and cellular process related genes expression (*WEE1*, *CCND1*, DG*KB, GPD1L2*, *BTRC* and *ATF2*) by competing miRNA targets, which could contributed more AFD in fat line. XR_001466431.2 is the exon sense-overlapping transcript of *C4H4ORF50* gene. Up to date, only few studies have reported the functions of *C4H4ORF50* gene (also named *C4orf50*). Its orthologs (human *C4orf50*) was highly expressed in brain tissues and related to Alzheimer’s disease and diabetes [59]. In our study, the *C4H4ORF50* gene was only expressed in abdominal fat tissues of fat line and could be regulated by the adjacent lncRNA XR_001466431.2 through ceRNA mechanism, which might supply the novel cognition of *C4H4ORF50* gene in chicken. Besides, XR_001466431.2 was identified to regulate cellular process and lipid metabolism related genes expression in the ceRNA network, which might be an important factor in adipogenesis of broilers. XR_003077610.1 is a natural antisense lncRNA nearby *FGF1* gene. As the fibroblast growth factor (FGF) gene family member, *FGF1* was highly expressed in adipose tissue of humans and mice, which implicated with adipocyte differentiation and adipose tissue remodeling [60–62]. In chicken, *FGF1* gene expression showed a positive correlation with intramuscular fat content in male thigh muscle, but a negative correlation in females. Although *FGF1* gene was not differentially expressed between the two lines, its cis-regulator XR_003077610.1 was only expressed in fat-line broilers. For lean-line broiler enriched lncRNA, XR_001464408.2 is the exon sense-overlapping transcript 4 of the *ALPK3* gene. Though the *ALPK3* gene was less reported in chickens, it has been comprehensively studied in the cardiomyopathy of mammals. *ALPK3* is a vital kinase in cell differentiation, and *ALPK3* knockout mice demonstrate both hypertrophic and dilated forms of cardiomyopathy [63, 64]. XR_210326.3 belongs to a bidirectional lncRNA oriented head to head to the *PAPD7* gene. PAPD7 is a cellular, noncanonical, poly (A) polymerase, mainly oligoadenylating the 3′ end of the noncoding RNA for exosome degradation [65]. As similar biological functions with the adjacent potential cis-targets [66], the XR_001464408.2, and XR_210326.3 might majorly affect cell differentiation and post-transcriptional modification. Besides, the two lncRNAs were found to regulate the *CH25H* and *AKR1B1L* genes respectively by competing in combination with the various miRNAs in the network. *CH25H* could indirectly inhibit lipids and cholesterol synthesis in broiler liver by regulating the activation of sterol regulatory element binding proteins [67]. Consistently, the lean-line broiler enriched XR_001464408.2 might up-regulated CH25H gene expression, which decreased the AFD in lean line. *AKR1B1L*, aldo-keto reductase (AKR) family 1 member B1-like, is implicated in carbohydrate metabolism and AKR1B1, as a homolog of *AKR1B1L*, impairs the fat storage by regulating the prostaglandin synthesis in human preadipocytes [68]. Compared to the fat line broilers, a higher *AKR1B1L* expression which is regulated by XR_210326.3 can be an important factor for less AFD in the lean line. These above obesity-related genes were identified to be regulated with the pivotal lncRNAs (e.g. XR_001468036.2, XR_003077610.1 and XR_001466431.2) in the ceRNA network, providing comprehensive insight into understanding the gene regulation mechanism in depositing fat in chicken.

For DE circRNAs, the functional enrichment of their host genes showed that the cellular processes and metabolism-related pathways and GO terms were mainly enriched. In subcutaneous adipogenesis in pigs, the DE circRNAs between the preadipocyte and mature adipocyte were found to be mainly involved in the cellular process [19]. The functional analysis of the DE circRNAs in the chicken adipocyte differentiation process identified a cellular process to be the most enriched GO term, and more lipid metabolism-related GO terms were also significantly enriched during the intramuscular and abdominal adipogenesis in chicken [27]. Our results were consistent with these studies, though we could not find significant enrichment levels for the lipid metabolism-related pathways (like “Adipocytokine signaling pathway”, “Insulin signaling pathway” and “MAPK signaling pathway”) in the present study (Additional file 1: Tables S7 and S9). We speculated that the main functional regulation of the DE circRNAs in the abdominal fat tissues between the fat- and lean-line broilers might differ from that in the adipocyte differentiation in vitro. To fully understand the underlying regulations of circRNA in fat deposition, the target miRNAs were further predicted for the identified DE circRNAs and a total of 314 miRNAs were identified as candidate targets for 17 DE circRNAs (Additional file 1: Table S5). Previously, our lab investigated the miRNA profiles of the preadipocytes in the 15th generation of NEAUHLF, and 33 miRNAs were identified, whose target genes were involved in adipocyte development and metabolism and were differentially expressed between the fat- and lean-line broilers (i.e. miR-31, miR-33, miR-206 et al.) [69]. Studies have proven gga-miR-33 to be closely related to the lipid metabolism and fatty acid oxidation in the chicken liver by regulating the *FTO*, *CROT,* and *HADHB* genes [70]. Our predicted miRNAs overlapped with the previous results, suggesting that the identified DE circRNAs could interact with these fat metabolism-related miRNAs to affect the deposition of abdominal fat. Our study described the comprehensive landscape of the lncRNAs and circRNAs of the abdominal fat tissues in the fat- and lean-line broilers and identified some candidate ncRNAs for depositing abdominal fat using bioinformatics analysis. However, further verifications and molecular experiments are needed for exploring the underlying mechanism of these ncRNAs in obesity.

## Conclusions

This study investigated the profiles of the lncRNAs and circRNAs in the abdominal fat tissues from the two groups of broilers with the variable ability of fat deposition. A total of 30 lncRNAs and 17 circRNAs were found to be significantly and differentially expressed in the abdominal fat between the fat- and lean-line broilers. Functional enrichments showed these DE ncRNAs to be mainly involved in the cellular processes and metabolism-related pathways. The target regulation analysis revealed the identified DE lncRNAs to possess strong regulatory correlations with the genes related to lipid metabolism and cellular process. In summary, the identified lncRNAs and circRNAs could serve as the vital regulators contributing to the divergent deposition of abdominal fat between the two lines of broilers. These candidates could be focused on our future study to completely investigate the occurrence and development of obesity in the broiler.

## Methods

### Animals and sampling

The animals and samples used in the present study were similar to those in our previous study [30]. Briefly, all the experimental broilers included 3 male fat-line broilers and 3 male lean-line broilers derived from the 23rd generation of the unique genetic obese-lean broiler lines, named Northeast Agricultural University broiler lines, which were divergently selected for the content of the abdominal fat (NEAUHLF). According to the previous breeding scheme, NEAUHLF now houses more than a 10-fold difference in the abdominal fat percentage (AFP: abdominal fat weight/body weight) between the two lines [24]. The abdominal fat tissues were sampled from six broilers and immediately frozen in liquid nitrogen. The samples were subsequently stored at − 80 °C until RNA was isolated.

### RNA isolation and library construction

The total RNA was extracted from the studied samples using the TRIzol reagent (Invitrogen Co., CA, USA) following the manufacturer’s instructions. The ribosomal-RNA was removed using the NEBNext rRNA Depletion Kit (New England Biolabs, Inc., Massachusetts, USA). Subsequently, the quality and integrity of the purified RNAs were evaluated using the NanoDrop ND-1000 (Thermo Fisher Scientific, Waltham, MA, USA) and agarose gel electrophoresis. The high-quality RNA samples were used for constructing the RNA libraries using the NEBNext® Ultra™ II Directional RNA Library Prep Kit (New England Biolabs, Inc., Massachusetts, USA). The eligible libraries obtained after quality control and quantification were sequenced on the Illumina Hiseq platform by the Cloudseq Biotech Inc. (Shanghai, China).

### Identification of the lncRNAs and circRNAs, and their functional enrichment

After harvesting the paired-end reads from the Illumina HiSeq 4000 sequencer, the quality control was performed by Q30. The low-quality reads were removed after 3′ adaptor-trimming, using the cutadapt software (v1.9.3) [71] while the high-quality trimmed reads were used for analyzing the lncRNAs and circRNAs. For identifying the lncRNAs, the high-quality reads were aligned to the chicken reference genome (GCF_000002315.6_GRCg6a) using the hisat2 software (v2.0.4) [72]. Then, the lncRNAs were identified under the guidance of the NCBI gtf gene annotation file, following these common criteria: 1) the transcripts annotation with known mRNAs and other types of non-coding RNAs (e.g. pseudogenes, pre-miRNA, tRNA, etc.) were removed; 2) the transcripts with exon numbers less than 2 and length shorter than 200 bp were filtered; 3) the protein-coding ability was assessed using four approaches, including the coding-non-coding index (CNCI), coding potential calculator (CPC), protein folding domain database (PFAM), and coding potential assessing tool (CPAT). The intersected results obtained represented the final identified lncRNAs. According to genomic location, the lncRNAs were classified into exon sense-overlapping lncRNA (the exon of the lncRNAs overlaps a coding transcript exon on the same genomic strand), intron sense-overlapping lncRNA (the lncRNA overlaps the intron of a coding transcript on the same genomic strand), intronic antisense lncRNA (the lncRNA overlap the intron of a coding transcript on the antisense strand), natural antisense lncRNA (the lncRNA transcribed from the antisense strand and overlapping with the exon of a coding transcript), bidirectional lncRNA (the lncRNA oriented head to head to a coding transcript within 1000 bp) and intergenic lncRNA (there are no overlapping or bidirectional coding transcripts near the lncRNA) (Additional file 2: Fig. S1) [73]. The FPKM was calculated as the expression profiles of lncRNA using the cuffdiff software (v2.2.1, part of cufflinks) [74, 75], and the fold change and *P-*value were calculated based on the FPKM. Subsequently, the differentially expressed (DE) lncRNAs were identified with the threshold of |log2FC| ≥ 1 and *P*-value < 0.05. The cis-targets of the lncRNAs were predicted based on the 50-kb upstream and downstream of their genomic position [19] and the trans-target mRNAs based on the Pearson correlation coefficient of the lncRNA-mRNA pairs exceeding 0.95 [76].

The high-quality reads were aligned to the reference genome/transcriptome for identifying the circRNA using the STAR software (v2.5.1b) [77], and the generated chimeric.out.junction files were subsequently used to detect circRNAs using the DCC software (v0.4.4) [78]. The circRNAs with junction reads over than one were considered as expressed. The circRNAs were classified based on the parent sequence location into exonic circRNA (sourced from the exons), intronic circRNA (sourced from the introns), intergenic circRNA (sourced from the intergenic regions), sense overlapping circRNA (sourced from the exons and intergenic regions), and antisense circRNA (sourced from the exons or introns in the antisense strand of the coding gene) (Additional file 2: Fig. S1) [79]. The data were normalized using the edgeR software (v3.16.5) and the DE circRNA was analyzed with the threshold of |log2FC| ≥ 1 and *P-*value < 0.05 [80]. Finally, the GO and KEGG pathways for the differentially expressed lncRNA targets and circRNA host genes were analyzed by R (version 4.0) [81].

### The validation of differential expressed lncRNAs and circRNAs

The five broilers of each line from the same generation of the NEAUHLF were selected randomly to perform the validate experiment. The total RNA of abdominal fat tissue was extracted using TRizol reagent (Invitrogen, CA, USA) following the manufacturer’s protocol. According to the recommended protocol of the PrimeScript™ RT reagent kit (Takara Bio Inc., Kusatsu, Japan), the probable genomic DNA in isolated RNA was firstly removed by gDNA Eraser followed by the reverse transcription reaction. Specific primers (Additional file 1: Table S11) for candidate RNAs (three each of the DE lncRNAs and DE circRNAs were randomly selected) were designed using Primer premier 5.0 software. The ABI QuantStudio™ 6 Flex Real-Time PCR System (Applied Biosystems, Foster City, CA, USA) was adopted to perform the qRT-PCR of candidate genes with FastStart SYBR Green Master (Roche, Mannheim, DEU). The reaction conditions were: an initial denaturation at 95 °C for 10 min, 40 cycles of 15 s at 95 °C and 1 min at 60 °C. C_t_ values of all genes were analyzed using QuantStudio™ Real-Time PCR Software v1.3 (Applied Biosystems). The housekeeping gene of TBP was used for normalization of C_t_ values. Gene expression comparison between the two lines was done using the 2^−ΔΔCt^ method.

### Construction of the co-expression and ceRNA network

To investigate the functions of pivotal lncRNAs, the co-expression network of the DE lncRNAs with their trans-target DE mRNAs was constructed using the Cytoscape software (version 3.6.1). The DE mRNAs (previously identified by our lab) [30] and annotated in lipid metabolism and cellular process-related pathways were used (Additional file 1: Table S3).

The expression of mRNA can be regulated by the lncRNA, which acts as a miRNA sponge that competitively binds with the miRNA [82]. Therefore, to investigate the competing regulations, a lncRNA-miRNA-mRNA ceRNA regulatory network was constructed by integrating the lncRNA-miRNA pairs and miRNA-mRNA pairs using the Cytoscape software. The 16 DE lncRNAs that were specifically expressed in either of the group and located in the cytoplasm were chosen in constructing the ceRNA network (Additional file 1: Table S1). The regulatory relationships of the lncRNA (predicted in the cytoplasm)-miRNA pairs and miRNA-mRNA pairs were predicted using online tools available on miRDB (http://www.mirdb.org/) with the threshold score of 70. The common miRNAs in the lncRNA-miRNA and miRNA-mRNA pairs were reserved for building the ceRNA network (Additional file 1: Table S10).

## Supplementary Information

**Additional file 1.**

## Data Availability

The datasets generated and/or analysed during the current study are available in the Sequence Read Archive (SRA) database on NCBI (accession number PRJNA657377; https://dataview.ncbi.nlm.nih.gov/object/PRJNA657377?reviewer=dm4k93jkenp6p5tbhmbh8iig8d).

## Abbreviations

lncRNA: Long non-coding RNA
circRNA: Circular RNA
DE: Differential expressed
ceRNA: Competing endogenous RNA
ncRNA: Non-coding RNA
AFD: Abdominal fat deposition
NEAUHLF: Northeast Agricultural University broiler lines, which were divergently selected for the content of the abdominal fat
AFP: Abdominal fat percentage
HDL: High density lipoprotein
HDL-C: High density lipoprotein cholesterol
lincRNA: Intergenic lncRNA
