# Supplementary material for: The landscape of the long non-coding RNAs and circular RNAs of the abdominal fat tissues in the chicken lines divergently selected for fatness
Source: BMC Genomics. 2022 Dec 1;23:790. doi: 10.1186/s12864-022-09045-y (PMC9714206; doi:10.1186/s12864-022-09045-y)
Supplement: Supplementary file 1 — Additional file 1. [file 12864_2022_9045_MOESM1_ESM.zip › Supplementary Information Legends.docx]

**Supplementary Information**

**Additional file 1: Table S1.** The identification of differentially expressed lncRNAs. **Table S2.** The identification of differentially expressed circRNAs. **Table S3.** The expression matrix of mRNAs used in the co-expression network. **Table S4.** Correlations between the lncRNAs and mRNAs. **Table S5.** The prediction of the potential miRNA targets for circRNAs. **Table S6.** The KEGG enrichment of the lncRNAs targets. **Table S7.** The KEGG enrichment of the circRNAs host genes. **Table S8.** The GO enrichment of lncRNAs targets. **Table S9.** The GO enrichment of circRNAs host genes. **Table S10.** The prediction of the lncRNA-miRNA and miRNA-mRNA pairs. **Table S11.** The primers information of validation experiment.

**Additional file 2: Fig. S1.** The statistics and schematic plot of various classified ncRNA. The classification and numbers of ncRNAs were displayed in the Figure. For lncRNAs (**A**), the green block represents mRNA, while the purple block represents lncRNA; the relative genomic position of the lncRNA and mRNA were displayed on a classification basis. For circRNAs (**B**), the green, blue and red blocks are exons of coding genes, while green and orange lines represent the intron and inter-genic areas; the circRNA classification is based on the sourced sequence. DE is differentially expressed. **Fig. S2.** The principal component analysis of the lncRNAs and circRNAs profiles in the abdominal fat between the fat- and lean-line broilers. The whole expression profiles of lncRNAs (**A**) and circRNAs (**B**) were analyzed. The fat- and lean-line broilers showed distinct expression patterns for the lncRNAs and circRNAs. **Fig. S3.** Validation of DE lncRNAs **(A)** and circRNAs **(B)** by using qRT-PCR. Log2FC in qRT-PCR equals -ΔΔC_t_ for each comparison (fat line vs. lean line). Average C_t_ value for each group was the means of samples in that group. The housekeeping gene of TBP was used for normalization of C_t_ values. ** means *P* value < 0.01. **Fig. S4.** The histogram of GO enrichments for the lncRNAs targets and circRNAs host genes. The top 10 most significant terms of each category were displayed, unless the enriched GO term count was less than 10, in which case, all the pathways were included in the plot. The Y-axis represented the name of enriched GO terms, where the X-axis represented the numbers of enriched lncRNAs targets (**A**) and circRNAs host genes (**B**). The adjusted *P*-value was set to 0.05, and terms of the same category were sorted by the enriched gene numbers.
